# Weekly Nowcasting of New COVID-19 Cases Using Past Viral Load Measurements

**DOI:** 10.3390/v14071414

**Published:** 2022-06-28

**Authors:** Athar Khalil, Khalil Al Handawi, Zeina Mohsen, Afif Abdel Nour, Rita Feghali, Ibrahim Chamseddine, Michael Kokkolaras

**Affiliations:** 1 Department of Genetics and Genome Sciences, School of Medicine, Case Western Reserve University, Cleveland, OH 44106, USA; axk1472@case.edu; 2 Clinical Research Unit, Rafik Hariri University Hospital, Beirut 2010, Lebanon; 3 Group for Research in Decision Analysis (GERAD), Montréal, QC H3T 1J4, Canada; khalil.alhandawi@mail.mcgill.ca (K.A.H.); michael.kokkolaras@mcgill.ca (M.K.); 4 Systems Optimization Lab, Department of Mechanical Engineering, McGill University, Montréal, QC H3A 0G4, Canada; 5 Department of Laboratory Medicine, Rafik Hariri University Hospital, Beirut 2010, Lebanon; zeinamohsen@outlook.com (Z.M.); rfeghali@ul.edu.lb (R.F.); 6 School of Engineering, The Holy Spirit University of Kaslik, Jounieh 446, Lebanon; afif.abdelnour@gmail.com; 7 Department of Radiation Oncology, Massachusetts General Hospital, Harvard Medical School, Boston, MA 02114, USA

**Keywords:** COVID-19, deep neural networks, viral load, Ct values, predictive modeling, machine learning, now-casting, statistical analysis

## Abstract

The rapid spread of the coronavirus disease COVID-19 has imposed clinical and financial burdens on hospitals and governments attempting to provide patients with medical care and implement disease-controlling policies. The transmissibility of the disease was shown to be correlated with the patient’s viral load, which can be measured during testing using the cycle threshold (Ct). Previous models have utilized Ct to forecast the trajectory of the spread, which can provide valuable information to better allocate resources and change policies. However, these models combined other variables specific to medical institutions or came in the form of compartmental models that rely on epidemiological assumptions, all of which could impose prediction uncertainties. In this study, we overcome these limitations using data-driven modeling that utilizes Ct and previous number of cases, two institution-independent variables. We collected three groups of patients (n = 6296, n = 3228, and n = 12,096) from different time periods to train, validate, and independently validate the models. We used three machine learning algorithms and three deep learning algorithms that can model the temporal dynamic behavior of the number of cases. The endpoint was 7-week forward number of cases, and the prediction was evaluated using mean square error (MSE). The sequence-to-sequence model showed the best prediction during validation (MSE = 0.025), while polynomial regression (OLS) and support vector machine regression (SVR) had better performance during independent validation (MSE = 0.1596, and MSE = 0.16754, respectively), which exhibited better generalizability of the latter. The OLS and SVR models were used on a dataset from an external institution and showed promise in predicting COVID-19 incidences across institutions. These models may support clinical and logistic decision-making after prospective validation.

## 1. Introduction

Coronavirus disease (COVID-19) was declared a pandemic by the World Health Organization (WHO) on March 2020 following the global spread of the underlying severe acute respiratory syndrome coronavirus-2 (SARS-CoV-2) [1,2]. The clinical outcomes of patients can range from an asymptomatic state to acute respiratory distress syndrome and multi-organ dysfunction that can lead to death. The most identified risk factors are age, sex, ethnicity, smoking status and other comorbidities such as cardiovascular disorders, chronic kidney diseases and diabetes [2,3]. SARS-CoV-2 can be transmitted via direct contact; within a distance of one meter through coughing, talking, or sneezing; or indirectly via infectious secretions from infected patients [3]. COVID-19 put a strain on the economy and caused the general well-being of the population to diminish due to the public health and social measures (PHSMs) employed to control it [4]. Now-casting models are used to infer the epidemic trajectory and make informed decisions about its severity and necessary actions needed to bring the epidemic under control. Information regarding the origin of the pathogen, serological assays, and social behavior, among other aspects, are used to inform now-casting models and provide situational awareness to policymakers [5]. Several works in the literature have used epidemiological size indicators such as the frequency of tests, fatalities and new confirmed cases to infer the pandemic trajectory [6,7]. This paper focuses on both the epidemic size and serological assays from a cross-sectional sample of patients to develop a now-casting framework. Predictive modeling and now-casting of epidemic trajectories can alert policymakers and health institutions about an increase in incidence rates. This allows sufficient time to use other detailed scenario models to proactively test and deploy various PHSMs [8,9,10,11]. Therefore, the transmission rate depends on the patient’s contagious stage, viral load, and the time of exposure between individuals [4].

RT-qPCR is a serological test that remains the gold standard for COVID-19 diagnosis [12]. It measures the first PCR cycle, denoted as the cycle threshold (Ct), at which a detectable signal of the targeted DNA appears [13]. The Ct value is inversely proportional to the viral load; a 3-point increase in Ct value equals a 10-fold decrease in the quantity of the virus’ genetic material [14]. Ct values were proposed to have potential prognostic value in predicting severity, infectiousness, and mortality among patients [15]. Ct values were also used to determine the duration an infected patient needs to quarantine [16,17]. A high Ct value (indicating a low viral load) is detected at early stages of the infection before the person becomes contagious and at the late stages when the risk of transmission is low [18]. The lowest possible Ct value is usually reported within three days of the onset of symptoms and coincides with peak detection of cultivable virus and infectivity that implies an increase in transmissibility by up to 8-fold [19]. Using viral load measurements, individuals with high viral load and mild symptoms can be identified as potential superspreaders [20]. Thus, early testing is highly recommended alongside isolation practices, to interrupt SARS-CoV-2 transmission [21].

Reverse-transcription quantitative polymerase chain reaction (RT-qPCR) is a serological test that remains the gold standard for COVID-19 diagnosis [12]. It measures the first PCR cycle, denoted as the cycle threshold (Ct), at which a detectable signal of the targeted DNA appears [13]. The Ct value is inversely related to the viral load; a 3-point increase in Ct value equals a 10-fold decrease in the quantity of the virus’ genetic material [14]. Ct values were proposed to have potential prognostic value in predicting severity, infectiousness, and mortality among patients [15]. Ct values were also used to determine the duration an infected patient needs to quarantine [16,17]. A high Ct value (indicating a low viral load) is detected at the early stages of the infection before the person becomes contagious and at the late stages when the risk of transmission is low [18]. The lowest possible Ct value is usually reported within three days of the onset of symptoms and coincides with peak detection of cultivable virus and infectivity implies an increase in transmissibility by up to 8-fold [19]. Individuals with high viral load and mild symptoms can be identified as potential superspreaders using viral load measurements [20]. Thus, early testing is highly recommended alongside isolation practices to interrupt SARS-CoV-2 transmission [21].

We believe that the use of Ct for now-casting has its merits since it is a commonly available parameter irrespective of demographics and is highly correlated with transmissibility and incidence rates [22,23]. A popular approach for now-casting the pandemic trajectory is to use Bayesian inference frameworks to inform the posterior distributions for susceptibleexposed-infectious-recovered (SEIR) models and the corresponding time-varying incidence rate [6,24]. These approaches are limited by the assumptions of the underlying SEIR models (homogeneous distribution of population traits and contacts). On the other hand, machine learning approaches make little to no assumptions about the underlying models describing the mechanics of the transmission and can potentially generalize better when the viral transmission is not completely understood and sufficient data is available.

We demonstrate the merits of this approach using a robust framework that leverages observed viral load measurements for time series now-casting of new COVID-19 cases for an upcoming 7-day time frame. The models are developed using a large cohort from a single cross-sectional virologic test center in Lebanon with a hold-out cohort for independent testing after the model is finalized. The Lebanese patient cohort used in this study is the largest and most consistent one in terms of serological assessment. This fact made the retrieved Ct values representative and reflective of the whole country.

Now-casting the pandemic trajectory can facilitate its containment and improves healthcare providers’ preparedness against new SARS-CoV-2 variants and the surge in new cases caused by them. Furthermore, now-casting the pandemic trajectory can support policymakers during the decline phase of the pandemic (e.g., when vaccination rates are high and herd immunity is beginning to take hold) to suggest the best time frame for relaxing current PHSMs without the risk of the pandemic relapsing.

## 2. Materials and Methods

### 2.1. Patient Population

We retrospectively collected de-identified data for all COVID-19 patients diagnosed at Rafik Hariri University Hospital (RHUH) in Lebanon between 1 March 2020, and 31 March 2021. Rafik Hariri University Hospital (RHUH) is the country’s leading institution for COVID-19 testing and treatment, and the collected cohort represents the nation’s COVID-19 trajectory well [25]. Ct values were retrieved from the electronic medical database of the hospital, considering the date of the first positive RT-qPCR test for each patient while disregarding any subsequent positive tests that may have resulted during follow-up visits. RNA extraction and RT-qPCR processing protocols were consistent over time and the used PCR machines had similar calibration. The daily COVID-19 confirmed case counts in Lebanon were obtained from the Lebanese Ministry of Public Health and Worldometers website [26,27]. This study was approved by the Ethical Committee of RHUH. Written informed consent was waived since the study is retrospective and the patients’ information was de-identified.

### 2.2. Study Design

We created 3 cohorts (discovery, testing, and independent validation) using a longitudinal data split. The discovery group (Group 1) was used for training and cross-validation [28] to tune the hyperparameters and calibrate the model weights. The testing group (Group 2) was reserved for testing the models’ performance and calculating the test error. This approach complies with the Transparent Reporting of a Multivariable Prediction Model for Individual Prognosis or Diagnosis (TRIPOD) [29], which represents a classification criterion for predictive modeling. It has four types of increasing reliability. Since the data was split randomly into discovery (Group 1) and test groups (Group 2) at the beginning of the study, the model is a TRIPOD type 2b. We used a third portion of the data (Group 3) for further independent validation of the models developed using the discovery group (Group 1). This third group of data is called the ‘unseen data group’. Such a longitudinal split of data enhances the model validation, making it between internal and external validation [29].

### 2.3. Predictive Modeling

**Figure 1:**
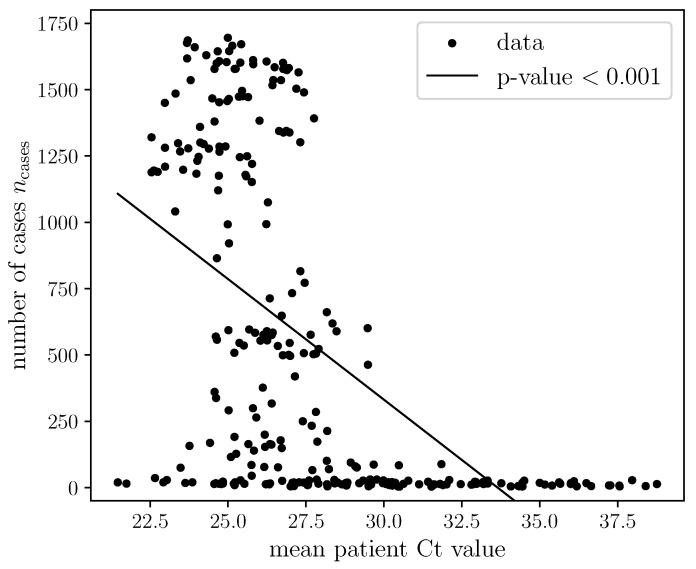
Scatter plot of biweekly mean Ct values and observed number of cases nationwide showing a clear negative value that is significant as given by *p*-value < 0.05.

We first identify the relevant input features needed by the models to predict epidemic trajectories in Lebanon using Spearman’s correlation test. We analyzed the association between the patients’ Ct values (Figure 1) and age (Figure S3a) with respect to the epidemic trajectory and only selected the features with p<0.05. Recent studies pointed out the case ascertainment rates may change over time (due to changes in PHSMs), resulting in biased Ct values [30]. The daily number of confirmed positive patients was plotted alongside the incidence rates in Lebanon to verify that this was not the case for the cohort used in this paper (see Section S1 and Figure S3b).

In addition to the previously mentioned features, the epidemic trajectory also depends on the past number of COVID-19 confirmed cases and is therefore aggregated with the input features during now-casting [5,7]. The period of time over which the input features and confirmed cases counts are aggregated is defined as the sliding window $T_{1}$. The input to all models is therefore a sequence of data over the past $T_{1}$ days.

The epidemic trajectory is given by a sequence of predicted case counts in the upcoming $T_{2}$ days and is fixed to 7 days throughout the study in this paper. The window size of 7 days on the epidemiological calendar was chosen due to its clinical relevance to health providers. Furthermore, other studies based on cross-sectional virological data have used the 7-day window size for now-casting pandemic trajectories [24]. We developed 6 different machine learning algorithms for now-casting the epidemic trajectory, which are described below.

#### 2.3.1. Recurrent Neural Network (RNN) Models

The first two models are built around recurrent neural networks (RNNs), which accommodate time-series data that are often temporarily correlated (i.e., the independent and identically distributed (i.i.d) assumption does not hold for time series data). This type of neural network can capture the temporal relationship between a decrease in Ct value and a subsequent (possibly delayed) rise in the number of cases. The RNN unit used in the models is the long short-term memory (LSTM) cell, which can capture long-term temporal effects and trends encoded by a long sequence of inputs and avoid the problem of vanishing gradients during backpropagation [31]. The LSTM has a cell for storing temporal data and gates to control data flow and capture long-term dependencies. Each gate is composed of a multilayer perceptron with $n_{\mathrm{hidden}}$ neurons [32]. We used stacked LSTM cells with several layers (given by $n_{\mathrm{layers}}$) in the RNN models to learn high-level feature representations (the interaction of Ct values with the past number of cases) and used a dropout probability $P_{\mathrm{dropout}}$ on all but the first layer to generalize better and avoid overfitting. Dropout arbitrarily excludes a number of hidden neurons from weight and bias updates during backpropagation to improve generalization performance [33]. Temporal information at time step $t_{i}$ of the *n*-th layer LSTM cell is represented by its hidden $h_{t_{i}}^{n}$ and cell states $C_{t_{i}}^{n}$.

**Figure 2:**
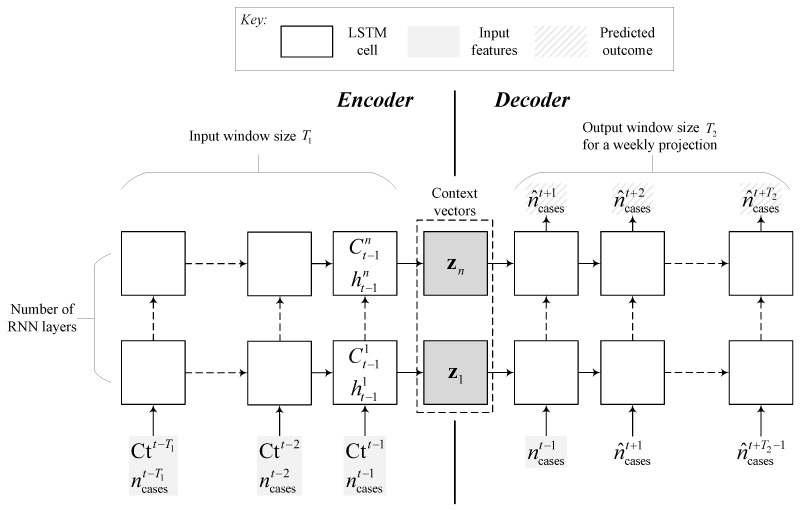
Structure of the sequence-to-sequence (S2S) model used for now-casting the weekly number of cases. The left side of the network is the encoder that uses past information on Ct and the number of cases to create context vectors used to initialize the hidden and cell states of the decoder LSTM cells.

The first model is given by a sequence-to-sequence (S2S) model commonly used in natural language processing (NLP) translation tasks. The model consists of an encoder RNN that accepts an input sequence of features of length $T_{1}$ and yields a context vector $z_{n}={\left[\begin{array}{cc} C_{t-1}^{n} & h_{t-1}^{n} \end{array}\right]}^{T}$, where t−1 is the final time step of the input series. The context vector is fed to a decoder that outputs a predicted sequence of length $T_{2}$ corresponding to the projected number of cases $n_{\mathrm{cases}}$. During training, the decoder uses its predictions ${\hat{n}}_{\mathrm{cases}}^{t_{i}}$ at time step $t_{i}$ as an input for the next the time step $t_{i+1}$. To speed up training, teacher forcing can be used to provide the actual value $n_{\mathrm{cases}}^{t_{i}}$ at time step $t_{i+1}$ instead of the decoder’s prediction with a probability $P_{\mathrm{teacher}}$ [34]. The architecture of the S2S model used in this paper is shown in Figure 2.

We also developed a second RNN model based on the stacked LSTM cells alone (i.e., the size of the input sequence $T_{1}$ must be equal to the size of the output sequence $T_{2}$) (Figure S4a). This model is called the stacked LSTM (SEQ) model.

The average number of predicted and actual number of cases for the next $T_{2}$ days is given by Equations (1) and (2), respectively

(1) $$\begin{array}{c} {\hat{\bar{n}}}_{\mathrm{cases}}=\frac{1}{T_{2}}\sum_{t_{i}=1}^{T_{2}}{\hat{n}}_{\mathrm{cases}}^{t_{i}}, \end{array}$$

(2) $$\begin{array}{c} {\bar{n}}_{\mathrm{cases}}=\frac{1}{T_{2}}\sum_{t_{i}=1}^{T_{2}}n_{\mathrm{cases}}^{t_{i}}. \end{array}$$

#### 2.3.2. Feedforward Neural Network (DNN) Model

We then developed a third model based on deep learning using feedforward neural networks. The feedforward neural network (DNN) model has several hidden layers ($n_{\mathrm{layers}}$) with several hidden neurons ($n_{\mathrm{hidden}}$) each. All layers had a dropout probability $P_{\mathrm{dropout}}$ and a rectified linear unit (ReLU) activation function (Figure S4b). All deep learning models were trained using the stochastic gradient descent algorithm ADAM with a learning rate $l_{\mathrm{rate}}$ and batch size $b_{\mathrm{size}}$ [35]. Early stopping was used on all deep learning models to avoid overfitting if no improvement in the validation error occurred after a certain number of epochs (given by the patience parameter $n_{\mathrm{patience}}$) [36].

#### 2.3.3. Regression Models

We developed three additional models that are not based on deep learning, namely a support vector machine regression (SVR) model [37], a gradient boosting machine (GBM) regression model [38], and a polynomial regression (OLS) model. Unlike deep learning models, these models do not yield a sequence of predictions for the next $T_{2}$ days. Instead, they compute a single value predicting the average number of confirmed COVID-19 cases for the next $T_{2}$ days (${\hat{\bar{n}}}_{\mathrm{cases}}$). This is because such models are primarily used for regression of univariate functions. This allows for a fair comparison with the models described in Section 2.3.1 and Section 2.3.2.

#### 2.3.4. Hyperparameter Tuning

**Figure 3:**
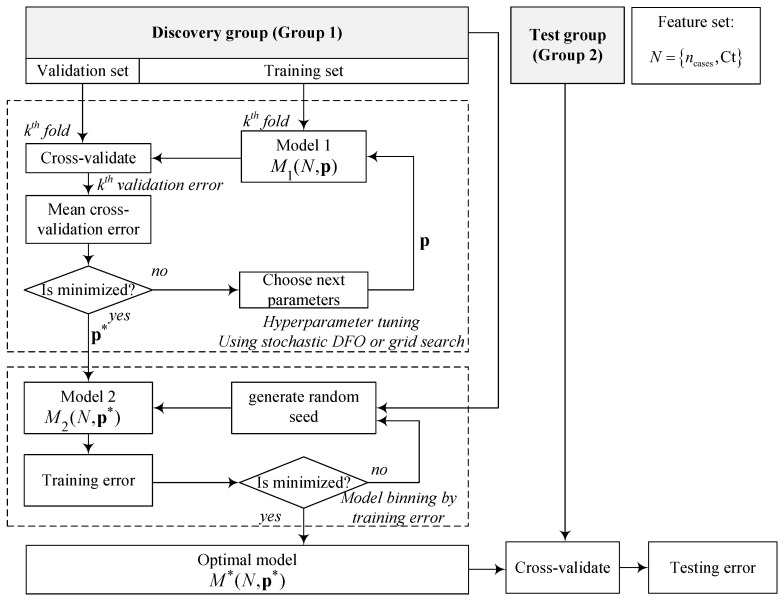
Cross-validation and hyperparameter determination scheme for model development. Following the discovery group (Group 1), the inner loop tuned the model’s hyperparameters by minimizing the average *k*-fold cross-validation error using a stochastic direct search algorithm or a grid search. The second loop (following tuning) generates several models randomly and bins them by training error. The best model with the lowest training error is tested on the test group to obtain the testing error.

The hyperparameters of each model (listed in and described in Table 1) were optimized using cross-validation on the discovery group (Group 1) only. The cross-validation consists of outer and inner loops (Figure 3).

The outer loop split (Group 1) into five groups and sent four into the inner loop for training the models and subsequent hyperparameter optimization with respect to the average k-fold cross-validation error [39]. The cross-validation error of each fold was calculated using the mean squared error (MSE) criterion on the predicted and actual average number of cases for the following $T_{2}$ days as shown in Equation (3).

(3) $$\mathrm{MSE}=\frac{1}{n_{\mathrm{days}}}\sum_{i=1}^{i=n_{\mathrm{days}}}{\left(n_{\mathrm{cases}}-{\hat{n}}_{\mathrm{cases}}\right)}^{2},$$

where $n_{\mathrm{cases}}$ and ${\hat{n}}_{\mathrm{cases}}$ are defined by Equations (1) and (2), respectively.

Several models used in this paper (S2S, SEQ, DNN, and GBM) involve random variables associated with the training algorithm (backpropagation and gradient boosting), which are often ignored in the literature of applied machine learning. Examples of these random variables include the initial value of learnable parameters (weights, biases, and decision tree parameters), dropout, and gradient descent step sizes. Fixing the random seed of these random variables could result in model bias.

We address this issue by randomly sampling different training runs during hyperparameter optimization and optimizing the mean cross-validation errors of all the sampled runs. We apply this approach to a grid search on the hyperparameter space to discern the sensitivity of the cross-validation error to the hyperparameters. We then use a stochastic derivative-free optimization (DFO) algorithm (stochastic mesh adaptive direct search (StoMADS)) to fine-tune the hyperparameters [40]. StoMADS is an extension of the mesh adaptive direct search (MADS) algorithm that automatically updates its estimates of a stochastic objective function (in this paper, the objective function is given by the cross-validation error) depending on the level of uncertainty in the current incumbent solution.

After obtaining the optimal model in the internal loop, we scored it using the outer loop data. We then performed a random draw to obtain 30 models using the tuned hyperparameters. These models were binned by training error and the top-performing model was stored and used to make predictions for the test group (Group 2).

We note that binning and sampling of the cross-validation error are unnecessary for the OLS and SVR models since their training is deterministic and does not involve random variables.

## 3. Results

### 3.1. Patient Population

The entire dataset included 23,185 patients with a median age of 37 years. We aggregated the individuals’ Ct into a sequence of daily mean Ct values. Group 1 contained 6296 patients admitted to RHUH between 2 March 2020 and 17 October 2020; Group 2 contained 3228 patients from 18 October 2020 to 30 November 2020, and the unseen group contained 12,097 patients from 1 December 2020 to 16 March 2021. All three groups have comparable median ages (34.0, 37.0, and 37.25 years, respectively). Group 1 was further split into five groups during model development for cross-validation: four training and one validation interchangeably.

**Figure 4:**
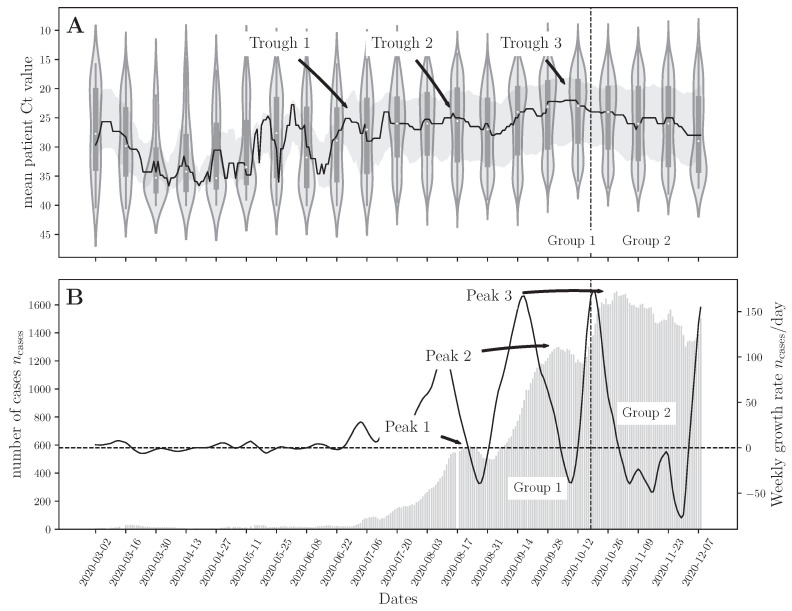
(**A**) Bi-weekly mean Ct values of RHUH patients. The solid line represents the median bi-weekly Ct values, and the gray shaded area represents the inter-quartile range (25–75 percentile) of the observed Ct values. (**B**) The grey bars show the weekly running average of the number of cases observed nationwide in Lebanon between 1 March 2020, and 7 December 2020 (the running average can be computed until 23 November). The solid black line represents the growth rate in the weekly number of cases.

Figure 4 shows the bi-weekly average Ct values observed and the corresponding number of cases in Lebanon nationwide for the period of time spanning groups 1 and 2 used in the model development phase. The entire dataset is provided in the Supplementary Material, Figure S1.

### 3.2. Correlation between the National Daily Number of COVID-19 Cases and Mean Ct

We observed a temporal delay between the incidence rate and the observed Ct values. For example, the trough in mean Ct values on 8 October 2020 (Trough 3 in Figure 4A) was followed by an increase in the number of cases, on 29 October 2020, with more than 1640 cases per day (Peak 3 in Figure 4B). This delay could be due to the time needed for population dynamics of disease transmission to take hold. Low Ct values indicate nascent infections circulating in the population that need time to reach the rest of the population. This observation has been reported by Hay et al. [24] using compartmental SEIR models to show that cross-sectional Ct observations with a low median value signal the growth phase of a pandemic (when case counts are still typically low). A similar trend was observed for case count peaks 1 and 2, which were superseded by median Ct troughs 1 and 2, respectively. This visual analysis of the data indicates that the median Ct value is temporally related to incidence.

We also investigate the relationship between the median Ct value and case counts using correlation analysis. We observed a clear inverse correlation between mean Ct and number of cases (*p* < 0.001), quantified by the Spearman correlation test (Figure 1). This indicates that the mean cross-sectional Ct value is an important feature for now-casting the pandemic trajectory.

### 3.3. Now-Casting the Epidemic Trajectories

**Figure 5:**
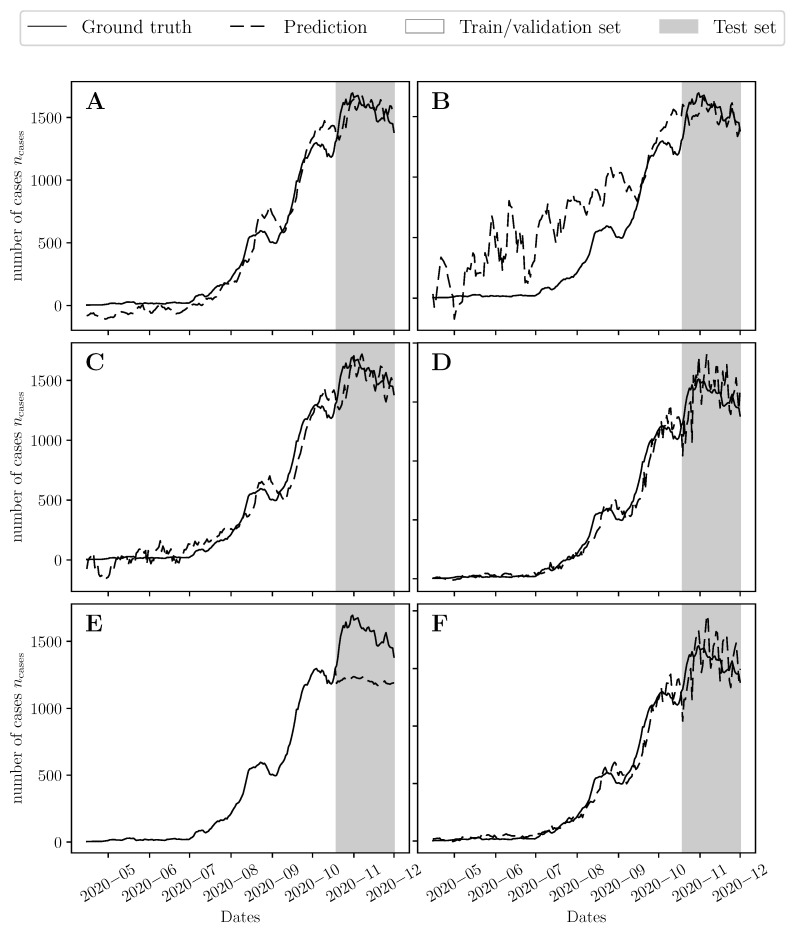
Predicted 7-day rolling average of daily number of cases on the unseen data set using (**A**) the sequence-to-sequence (S2S) model, (**B**) the stacked LSTM (SEQ), (**C**) The feedforward neural network (DNN), (**D**) The support vector machine regression (SVR) model, (**E**) The gradient boosting machine (GBM), and (**F**) the polynomial regression (OLS) model. All models were tuned using the cross-validation error of the discovery set. The grey shaded region represents the test data set (Group 2) used to test the models’ performance.

We developed six types of predictive models for now-casting the COVID-19 epidemic trajectory in Lebanon using the data in the discovery group (Group 1). The optimal hyperparameters for each model are listed in Table 1. Early stopping terminated the backpropagation algorithm at 31, 1, and 4 epochs for the S2S, SEQ, and DNN models, respectively. All models except the GBM had an optimal input window size $T_{1}$ of 6 days. This implies that an aggregate measure of cross-sectional Ct values and past incidence rates over the last 6 days could be used to now-cast the expected number of positive COVID-19 cases over the following 7 days. The models developed using Group 1 were used to now-cast the trajectory from 18 October 2020 to 30 November 2020 (Group 2) (Figure 5). The models were then retrained in Groups 1 and 2 using the hyperparameters in Table 1 and used to now-cast the epidemic trajectory after 1 December 2020 (Group 3). Table 2 lists the MSE error for the predicted trajectories on Groups 2 and 3 (see Table 2 footnote).

The RNN models (S2S and SEQ) performed well on Group 2 (MSE of 0.025 and 0.027, respectively), followed by the DNN model with an MSE that is two-fold larger (0.042). The OLS and SVR had an MSE that is four-fold larger than that of the RNN models (0.090 and 0.083, respectively). The GBM was heavily biased and did not generalize well on Group 2 (MSE of 0.326). The training error for the RNN models was higher than that of the parametric models (OLS and SVR) due to the regularization performed by the early-stopping criterion to avoid overfitting. Movie S1 shows an example training run of the S2S model with arbitrary hyperparameters, where early stopping helped avoid overfitting.

The MSE error of the SVR, OLS, and DNN models was comparable on the unseen data group (MSE values of 0.168, and 0.160, and 0.255, respectively). The SEQ and S2S performed worse on the unseen group, implying that simpler models perform better on the unseen group due to the limited number of data points available for training and hyperparameter tuning. Deep learning models generally excel when a large dataset is available for model development and has been reported by several studies in the literature [41,42].

To verify this, the RNN models were re-developed using both Groups 1 and 2 for training, hyperparameter tuning and validation. The generalization performance improved significantly, bringing the MSE error down from 0.571 to 0.106 for the S2S model (Table S1). This implies that the RNN models generalize better when more training data is available (see Supplementary Material Section S3). If limited data are available (at the start of a pandemic), simpler models can provide better generalization performance. We deployed the models developed on the combined dataset (Groups 1 and 2) as a web application to facilitate prospective validation in the future [43].

## 4. Discussion

Host viral load and the resultant Ct values have been widely proposed to evaluate the progression of SARS-CoV-2 infection and address patients’ contagiousness [44]. Mathematical modeling has been widely used for predicting the course of the COVID-19 pandemic. These prediction models were developed based on the applied intervention measurements and the population behavioral fluctuations, including social distancing and mask-wearing [45]. The COVID-19 reproduction number (R0), defined as the average number of naive individuals a patient can infect, has a mean estimate of 3.28 and could range from 1.4 to 6.49 [46]. Although R0 can widely vary by country, culture, and stage of the outbreak, it has been used to justify the need for community mitigation strategies and political interventions [47]. So far, only few advanced and more recent models have evaluated the disease spread based on viral kinetics and serological assays (such as RT-qPCR tests) [22,24]. Furthermore, these studies focused on serological assays or pandemic size indicators (such as R0 and incidence rates) in isolation without combining the two. This paper utilizes both past incidence rates and serological viral load measurements to now-cast the pandemic trajectory.

Hay et al. [24] used Bayesian inference to predict the growth rate in the daily number of COVID-19 cases as a function of Ct values [24]. They showed that the population-level Ct distribution is strongly correlated with growth rate estimates of new infections in MA, USA. They estimated R0 and growth rate by using observations of Ct values to inform priors on key viral kinetics parameters (such as the viral load wane rate, and Ct at peak viral load and the pandemic trajectory (daily probability of infection is used as a proxy for the trajectory). The prior on the pandemic trajectory is assumed to come from a Gaussian process that makes no assumptions regarding the evolution of the trajectory as more Ct observations are made. We have used the Gaussian process regression model to predict the pandemic trajectory using the RHUH cross-sectional patient cohort (see Supplementary Material Section S4). The advantage of such models is that they are highly interpretable as they estimate the viral kinetics model parameters that are most likely to give rise to the observed Ct values [48]. This provides useful information about the virulence and severity of the pathogen. However, such models make assumptions about the likelihood used to update the priors. These assumptions limit the predictive capability of the model if any of these assumptions (such as the viral kinetics models) do not hold in reality, potentially resulting in poor generalization performance. This is the case when a different clade of viruses takes hold. Another dataset from Bahrain demonstrated the effectiveness of Ct in predicting the epidemiological dynamics of COVID-19 [23]. However, the study did not consider the interaction between different features (i.e., number of positive cases and Ct), nor does it consider temporal effects observed in epidemics.

In comparison, the presented data-driven approach of inferring the epidemic trajectory using past case counts and Ct values makes very little assumptions about the pandemic trajectory and viral kinetics models that gave rise to the observed Ct values. This has the benefit of potentially generalizing to a wide range of scenarios. To prove this, we used all the models developed in this paper using Group 1 (Figure 5) to infer the case counts in the state of Massachusetts using the patient cohort of Brigham andWomen’s Hospital (BWH) provided by Hay et al. [24] (Figure S9A). Most models captured the underlying trend except for GBM and the stacked LSTM (SEQ) models (Figure S10). SVR performed the best on this dataset (Figure S9B). However, further prospective validation is needed in the future to ensure that these models can generalize to different testing centers and reject disturbances in Ct values due to sample collection and handling methods.

The inferred trajectories for the state of Massachusetts (15 April 2020–15 December 2020) and Lebanon (1 December 2020–31 March 2021) show that simple machine learning models (such as OLS and SVR) perform well with limited training data (when developing the models using data from Group 1 only). Deep learning models begin to outperform such models when including more data in the development set (Groups 1 and 2) to infer the trajectory in Lebanon (Figure S5). Although the outcomes of this study favored simpler regression models, their simplicity provides an advantage in terms of interpretability [48].

The dataset used in this study contained fluctuations that allowed us to extract the Ct temporal effect on the trajectory of the pandemic. Since the data came from a single institution, the fluctuations are likely to be signals in the data rather than noise. The significant changes in the Ct values mirrored the well-recognized political, economic, and social turning points that happened in Lebanon during the pandemic. These incidences impacted the population’s behavior towards COVID-19 in a consistent and well-defined manner, allowing us to track and correlate these changes with the variation in the mean Ct values and subsequently the disease spread. The early reported high mean Ct values and the low number of COVID-19 cases in Lebanon between March 2020 and June 2020 co-occurred with a strictly imposed lockdown and a harsh awareness campaign executed by local media platforms [25]. In comparison, the sharp rise in COVID-19 cases and the decrease in mean Ct values upon diagnosis were detected after releasing the first national lockdown in July, which occurred with a significant shifting of local media attention towards the economic crisis peaking in the country. Yet, the highest jump in the number of national COVID-19 patients and the sharpest drop in Ct values were reported after the explosion of Beirut’s port in August 2020, which was classified among the most significant chemical explosions in history [49]. The devastating effects of the explosion amplified the country’s pre-existing social, economic, and health challenges, causing a significant increase in the COVID-19 positivity rate in September and November 2020, which had reached 13.9% [49,50]. The consequences of this explosion shifted the residents’ attention away from proper precautions. This was reflected by the sharp decrease in the mean Ct values indicating less responsible behavior and a delay in diagnosis time among suspected patients, which resulted in a subsequent increase in SAR-CoV-2 spread among individuals. These events caused three significant peaks in the number of cases and three drops in mean Ct. We trained the models on two of these peaks and tested its ability to detect the third peak using the unseen data. Thus, the training and validation errors reflect the models’ robustness against unexpected events.

The detected inverse relationship between Ct values and the number of national COVID-19 positive cases reflects population dynamics of transmission and demonstrates the temporal significance of Ct values. The results emphasized the importance of early testing when the patient’s viral load and infectivity are low to prompt isolation practices and thus, suppress the national spread of the virus. The models were able to predict the upcoming one-week expected number of national COVID-19 cases based on a commonly available diagnostic measurement, the Ct value. This shows that viral load measurements are a rigid input that can enhance the outcomes of disease forecasting models. Interestingly, this model is still valuable among vaccinated patients as these patients were shown to have a similar viral load pattern as unvaccinated patients and thus, can efficiently transmit the disease in the same manner upon infection [51]. Ultimately, the data promoted incorporating Ct values with other epidemiological variables and patient demographics to predict new COVID-19 waves and to study epidemic behaviors. The models in this paper could be extended to now-cast other contagious viral diseases that are diagnosed by qPCR, provided that sufficient training data is available (at least one wave of the viral disease has been observed).

This study is limited to a single-institution cohort. Although the cohort represents the national number of cases, and the model’s variable (Ct) is country-independent, a prospective validation on multi-institutional data is needed before translation. To facilitate this process, we have hosted the models on a web interface to be used in future studies that compare the predicted and observed number of cases [43]. Another limitation is the inability of the model to compare the effect of preventative policies such as lockdowns and quarantining. The model provides an alert when the number of cases is about to rise significantly, allowing more informed triage decisions and better allocation of medical resources during the pandemic. However, it does not provide guidance on what measures could best control an upcoming peak. Mechanistic models, on the other hand, such as individualbased models (IBMs), can provide such insights but their application is limited to a much smaller population size due to computational cost [52,53,54,55]. A future study could focus on combining IBMs with viral load models such as those developed by Hay et al. [24] to estimate Ct values for a cross-section of the population and use them to retrain the models developed in this paper to now-cast the trajectory under different intervention policies [24].

## 5. Conclusions

This study is a first attempt at combining serologic assays from a representative cross-sectional patient cohort with epidemiological indicators such as incidence rates and infection size to now-cast the number of nationwide positive COVID-19 cases in a specific region [5]. This was motivated the premise that SARS-CoV-2 spread is highly dependent on the individual viral dynamics. The models used in this paper showed the merits of this approach using observations of Ct values and historical infection data from Lebanon in a now-casting framework. The modeling framework relied on multiple machine learning algorithms that make little assumptions about population and transmission dynamics. The patient cohort revealed that the evolution of the viral load mirrored the growth of positive national cases in the country. Low mean Ct values were followed by a large number of national positive COVID-19 cases and vice versa, in line with similar observations in the literature [22,24]. This finding is also supported by applying the machine learning models in this paper to the BWH dataset provided by Hay et al. [24]. To account for the effect of social interactions that could occur a few days before and after testing, we used a sequence of daily mean Ct values across multiple machine learning algorithms. We trained the models on a training dataset and independently validated them on unseen data forming TRIPOD type 2b models [29]. The training process utilized a cross-validation approach combined with a state-of-the-art stochastic direct search for hyperparameter tuning to prevent model over-fitting [56]. The sequence-to-sequence (S2S) model had the best accuracy when a large amount of data was used for its development, while the support vector machine regression (SVR) model provided better accuracy with limited development data as given by the MSE criterion. Since the models were trained and validated on datasets from different time periods, they have the potential to extend to future data. In addition, since the variables used for prediction (Ct values) are not specific to the institution from which the data were acquired, the models are ready to undergo a prospective and external validation in the future. This will form a TRIPOD 4 study, which is recommended to translate the model to practice to now-cast a 7-day forward number of cases based on recently reported Ct values.

## Figures and Tables

**Figure 6:**
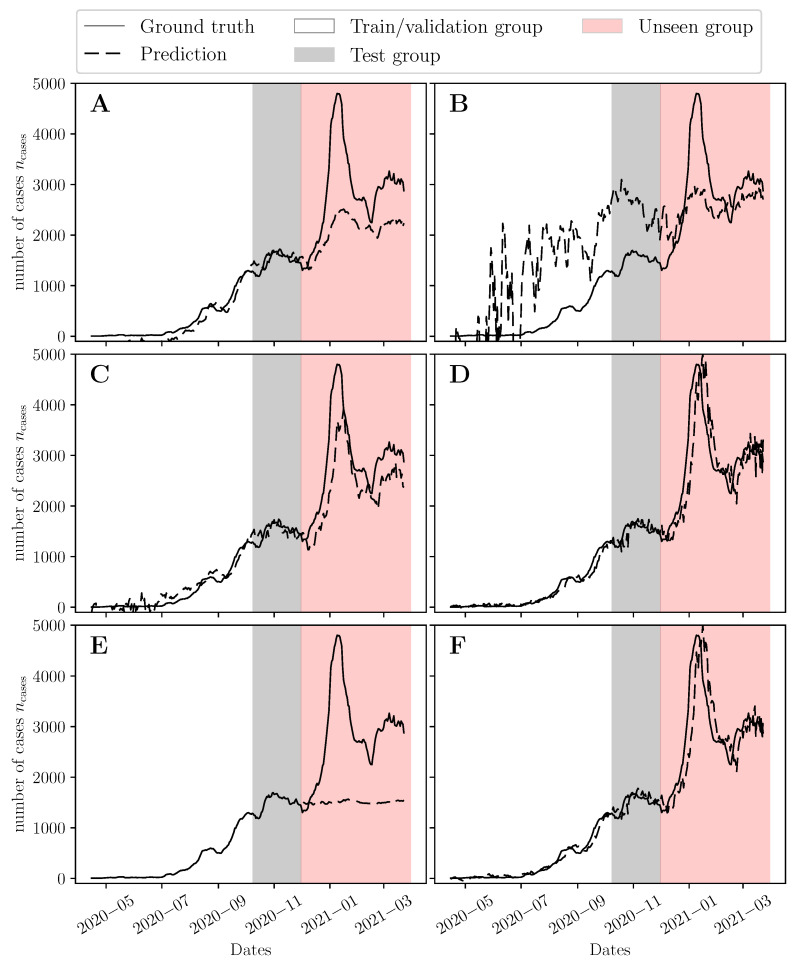
Predicted 7-day rolling average of daily number of cases on the unseen data set using (**A**) the sequence-to-sequence (S2S) model, (**B**) the stacked LSTM (SEQ), (**C**) The feedforward neural network (DNN), (**D**) The support vector machine regression (SVR) model, (**E**) The gradient boosting machine (GBM), and (**F**) the polynomial regression (OLS) model. All models were tuned using the validation error of the discovery set. The grey shaded region represents the test data set (Group 2) used to test the models’ performance. The models were retrained using both the discovery and test data sets and subsequently used to infer the number of cases in the unseen data set (the red shaded region).

**Table 1:** Optimal hyperparameters of different models.

Hyperparameter	Symbol	Value	Possible Values
Sequence-to-sequence model (S2S)			
Sliding window size	$T_{1}$	6	1–40
Number of hidden neurons	$n_{\mathrm{hidden}}$	1500	1–2500
Probability of dropout	$P_{\mathrm{dropout}}$	0.8	0.0–0.9
Number of hidden layers	$n_{\mathrm{hidden}}$	2	1–5
Teacher forcing probability	$P_{\mathrm{teacher}}$	0.3	0.0–0.9
Learning rate	$l_{\mathrm{rate}}$	$1\times 10^{-4}$	$1\times 10^{-5}$–$1\times 10^{-2}$
batch size	$b_{\mathrm{size}}$	32	4–128
best epoch	$n_{\mathrm{epochs}}^{\mathrm{best}}$	31	1–$n_{\mathrm{epochs}}$
Sequence completion model (SEQ)			
Number of hidden neurons	$n_{\mathrm{hidden}}$	2500	1–2500
Probability of dropout	$P_{\mathrm{dropout}}$	0.8	0.0–0.9
Number of hidden layers	$n_{\mathrm{hidden}}$	3	1–5
Learning rate	$l_{\mathrm{rate}}$	$1\times 10^{-4}$	$1\times 10^{-5}$–$1\times 10^{-2}$
batch size	$b_{\mathrm{size}}$	64	4–128
best epoch	$n_{\mathrm{epochs}}^{\mathrm{best}}$	1	1–$n_{\mathrm{epochs}}$
Deep neural network (DNN)			
Sliding window size	$T_{1}$	6	1–40
Number of hidden neurons	$n_{\mathrm{hidden}}$	1000	1–2500
Probability of dropout	$P_{\mathrm{dropout}}$	0.9	0.0–0.9
Number of hidden layers	$n_{\mathrm{hidden}}$	1	1–5
Learning rate	$l_{\mathrm{rate}}$	$1\times 10^{-3}$	$1\times 10^{-5}$–$1\times 10^{-2}$
batch size	$b_{\mathrm{size}}$	4	4–128
best epoch	$n_{\mathrm{epochs}}^{\mathrm{best}}$	4	1–$n_{\mathrm{epochs}}$
Support vector machine regression (SVR)			
Sliding window size	$T_{1}$	6	1–40
Ridge factor	λ	$1\times 10^{-4}$	$1\times 10^{-3}$−1.0
Margin of tolerance	ϵ	$1\times 10^{-2}$	$1\times 10^{-3}$–1.0
Stopping criteria tolerance	$\epsilon_{\mathrm{tol}}$	0.1	1–5
Learning rate	$l_{\mathrm{rate}}$	$1\times 10^{-5}$	$1\times 10^{-5}$–$1\times 10^{-2}$
Gradient boosting machine (GBM)			
Sliding window size	$T_{1}$	36	1–40
Subsample fraction	$f_{\mathrm{sample}}$	0.8	0.1–1.0
Maximum portion of features	$f_{\mathrm{features}}$	0.1	0.1–1.0
Decision tree maximum depth	*D*	7	1–5
Learning rate	$l_{\mathrm{rate}}$	0.01	$1\times 10^{-5}$–$1\times 10^{-2}$
Maximum number of boosting stages	$n_{\mathrm{stages}}$	5000	50–5000
Polynomial regression (OLS)			
Sliding window size	$T_{1}$	6	1–40
Ridge factor	λ	1.0	$1\times 10^{-3}$–1.0
Degree	$n_{\mathrm{degree}}$	1	1–5
Common fixed parameters			
Output window size (all models)	$T_{2}$	7	1–40
Maximum number of epochs (all models)	$n_{\mathrm{epochs}}$	5000	
Kernel (SVR)		linear	
Early stopping patience (S2S, SEQ, DNN)	$n_{\mathrm{patience}}$	200	
Optimizer (S2S, SEQ, DNN)		Adam	

The tuned hyperparameters of each model are reported underneath it. The fixed hyperparameters are reported at the bottom of the table.

**Table 2:** Training and testing errors given by mean squared error (MSE) of different models constructed using different feature sets.

Model	Figure 5		Figure 6	
	Train Error	Test Error	Train Error	Unseen Error
	Group 1	Group 2	Groups 1,2	Group 3
Sequence-to-sequence (S2S)	0.02462	0.02504	0.01309	0.57112
Stacked LSTM (SEQ)	0.38373	0.02724	0.78142	0.32584
Feedforward neural network (DNN)	0.02223	0.04179	0.00919	0.25547
Support vector machine regression (SVR)	0.01362	0.08347	0.00518	0.16754
Gradient boosting machine (GBM)	2.316 × 10${}^{-6}$	0.32589	2.316 × 10${}^{-6}$	1.44463
Polynomial regression (OLS)	0.01335	0.08954	0.00459	0.15954

The MSE in Equation (3) is computed using the standardized value of the predictions by normalizing them using the mean and standard deviation of all the daily number of cases (*n*_cases_) given by 463.8 and 597.0, respectively.

## Supplementary Materials

The following are available at https://www.mdpi.com/article/10.3390/v14071414/s1, Figure S1: (A) Bi-weekly mean Ct values of RHUH patients. The solid line represents the median bi-weekly Ct values, and the gray shaded area represents the inter-quartile range (25–75 percentile) of the observed Ct values. (B) The grey bars show the weekly running average of the number of cases observed nationwide in Lebanon between 1 March 2020, and 31 March 2021 (the running average can be computed until March 16). The solid black line represents the growth rate in the weekly number of cases. Figure S2: (A) Bi-weekly mean age of RHUH patients. The solid line represents the median patient age, and the gray shaded area represents the inter-quartile range (25–75 percentile) of the observed patient ages. (B) Bi-weekly mean number of confirmed positive RHUH patients. (C) The grey bars show the weekly running average of the number of cases observed nationwide in Lebanon between 1 March 2020, and 30 December 2020 (the running average can be computed until December 07). Figure S3: (A) Scatter plot of biweekly mean patient age and observed number of cases nationwide showing no significant relationship as given by *p*-value > 0.05 (B) Scatter plot of biweekly number of confirmed positive RHUH patients and observed number of cases nationwide showing a clear positive value that is significant as given by *p*-value < 0.05. Figure S4: Model architecture of the (a) stacked LSTM (SEQ) and the (b) feedforward neural network (DNN) models. Figure S5: Predicted 7-day rolling average of daily number of cases on the unseen data group using (A) the sequence-to-sequence (S2S) model, (B) the stacked LSTM (SEQ), (C) The feedforward neural network (DNN), (D) The support vector machine regression (SVR) model, (E) The gradient boosting machine (GBM), and (F) the polynomial regression (OLS) model. All models were tuned using the validation score of the combined discovery and test sets (Groups 1 and 2). The grey shaded region represents the unseen data group used to test the models’ performance. Figure S6: Illustration of variance in (a) training errors on the discovery group (Group 1) and (b) test errors on the test group (Group 2) for different models. The errors were calculated using the MSE of the predicted and actual trajectories shown in Figure 5. The green triangles represent the mean error of 30 independent training runs for each model type. The orange lines represent the median error. Figure S7: Illustration of variance in (a) training errors on combined discovery and test groups (Groups 1 and 2) and (b) the test errors on the unseen group (Group 3) for different models. The errors were calculated using the MSE of the predicted and actual trajectories shown in Figure S5. The green triangles represent the mean error of 30 independent training runs for each model type. The orange lines represent the median error. Figure S8: Incidence rate and pandemic trajectory predictions using the predictive framework developed by Hay et al. [24] (A) shows the cross-sectional Ct samples (violin plots) and smoothed average (solid blue line) obtained from RHUH throughout the pandemic in Lebanon. (B) Posterior distribution of relative probability of infection by date from a Gaussian process (GP) model fit to all observed Ct values (ribbons show 95% and 50% credible intervals, line shows posterior median). The y-axis shows relative rather than absolute probability of infection, as the underlying incidence curve must sum to one. The grey bars show the true case counts in Lebanon from the start of infection and have been normalized by the total number of cases observed in Lebanon throughout the observation time period shown (1 March 2020 through 30 November 2020). Figure S9: Incidence rate and pandemic trajectory predictions using the support vector machine regression (SVR) model (A) shows the cross-sectional Ct samples (violin plots) and smoothed average (solid blue line) obtained from Brigham and Women’s Hospital (BWH) throughout the pandemic in Massachusetts. (B) Predicted pandemic trajectory of the SVR model fit to all observed Ct values. The grey bars show the true case counts in Massachusetts from the start of infection. Figure S10: Predicted 7-day rolling average of the daily number of cases in Massachusetts predicted using (A) the sequence-to-sequence (S2S) model, (B) the stacked LSTM (SEQ), (C) The feedforward neural network (DNN), (D) The support vector machine regression (SVR) model, (E) The gradient boosting machine (GBM), and (F) the polynomial regression (OLS) model. The Ct values used in inference were obtained from Brigham and Women’s Hospital (BWH) [24]. Table S1: Training and testing errors given by mean squared error (MSE) of different models constructed using the combined discovery and test sets (Groups 1 and 2). Table S2: Optimal hyperparameters of models developed using combined discovery and test groups (Groups 1 and 2).
