# Supplementary material for: Weekly Nowcasting of New COVID-19 Cases Using Past Viral Load Measurements
Source: Viruses. 2022 Jun 28;14(7):1414. doi: 10.3390/v14071414 (PMC9317659; doi:10.3390/v14071414)
Supplement: Supplementary file 1 [file viruses-14-01414-s001.zip › viruses-1734735-supplementary.pdf]

Supplementary Materials: Weekly Nowcasting of New COVID-19 Cases Using Past Viral Load Measurements

S.1. Other patient cohort data

In this section, we graphically depict the unseen data (Group 3) used for independent validation of the models that were developed in Section 3.3. Figure S1 shows the unseen data from December 01, 2020 to March 16, 2021. The observation that low cycle threshold (Ct) values coincide with high case counts as was observed in Section 3.2 still holds for Group 3. Other features such as mean patient age and daily number of confirmed positive patients that were not used in model development are shown in Figure S2. Visual inspection of Figures S2A and C shows that there is no significant relationship between the mean age of a cross-sectional patient cohort and the incidence rates. This is confirmed by the correlation test done in Figure S3a. On the other hand, visual inspection of Figures S2B and C shows a significant correlation between the daily number of confirmed positive patients and the incidence rates. This is confirmed in Figure S3b. This significant correlation indicates that the patient cohort is indeed representative of the population from which it is derived and can be used to derive valid epidemiological indicators for this study. This ascertains the fact that consistent testing practices at Rafik Hariri University Hospital (RHUH) were followed throughout the study period (March 01, 2020 through March 16, 2021).

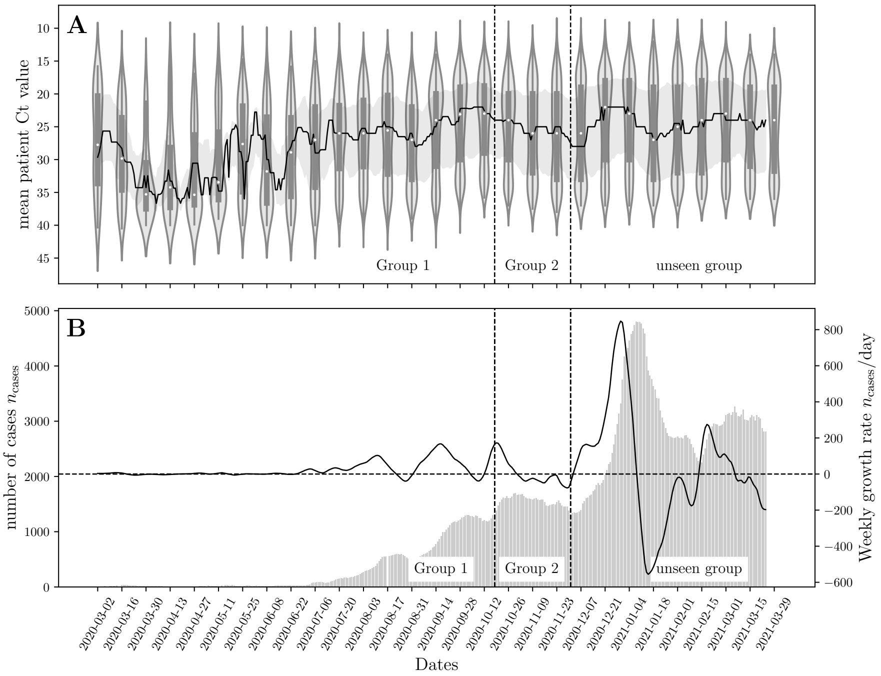

Figure S1. (A) Bi-weekly mean Ct values of RHUH patients. The solid line represents the median bi-weekly Ct values, and the gray shaded area represents the inter-quartile range (25-75 percentile) of the observed Ct values. (B) The grey bars show the weekly running average of the number of cases observed nationwide in Lebanon between March 1, 2020, and March 31, 2021 (the running average can be computed until March 16). The solid black line represents the growth rate in the weekly number of cases.

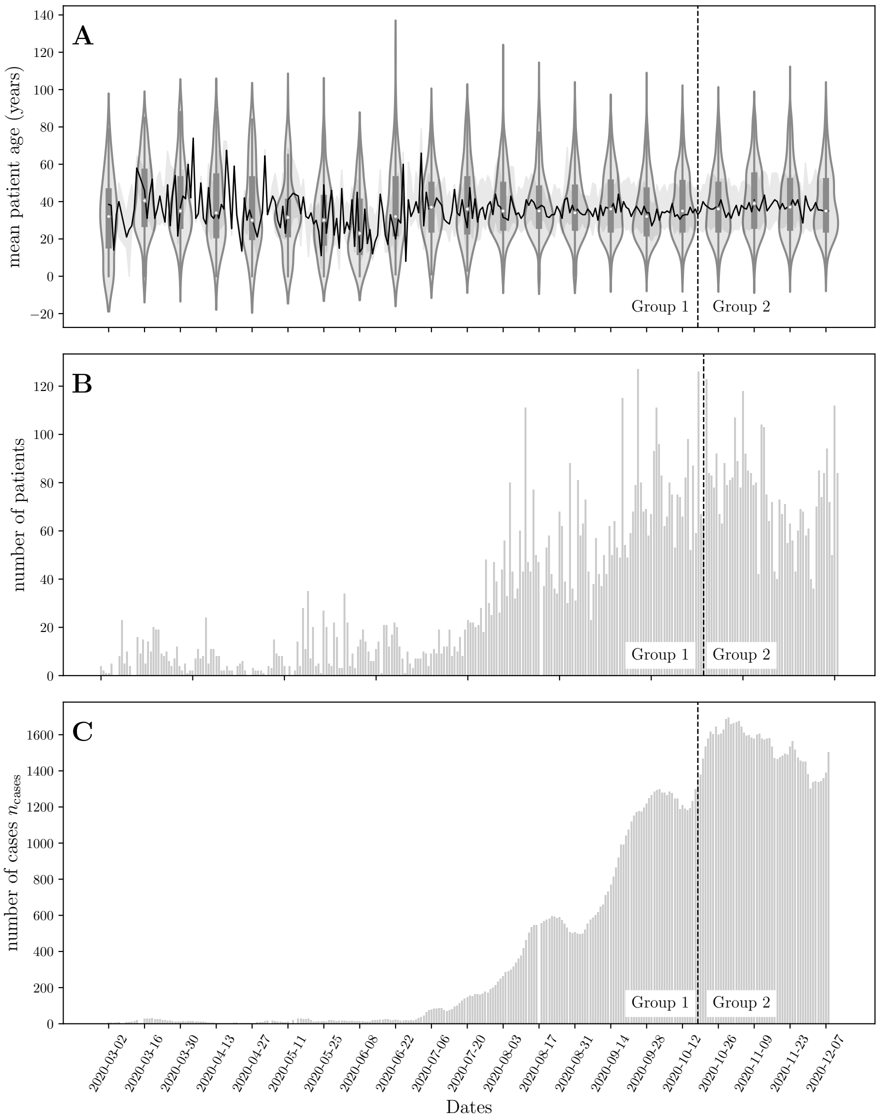

Figure S2. (A) Bi-weekly mean age of RHUH patients. The solid line represents the median patient age, and the gray shaded area represents the inter-quartile range (25-75 percentile) of the observed patient ages. (B) Bi-weekly mean number of confirmed positive RHUH patients. (C) The grey bars show the weekly running average of the number of cases observed nationwide in Lebanon between March 1st, 2020, and December 30, 2020 (the running average can be computed until December 07).

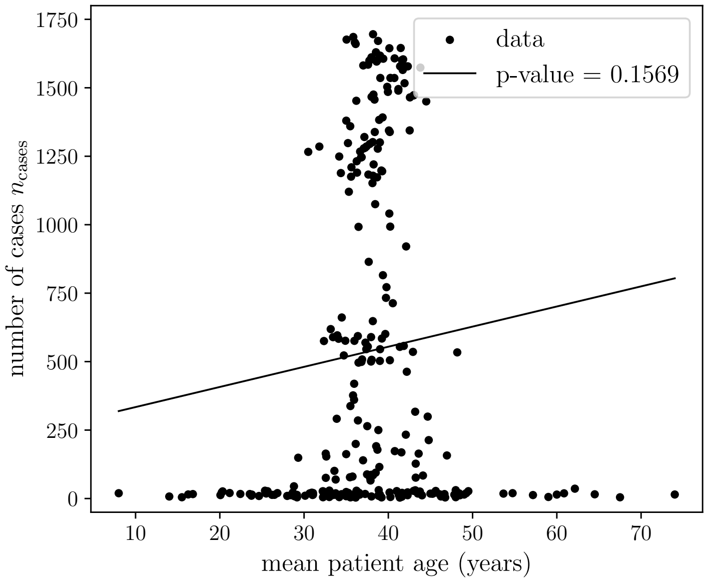

(a)

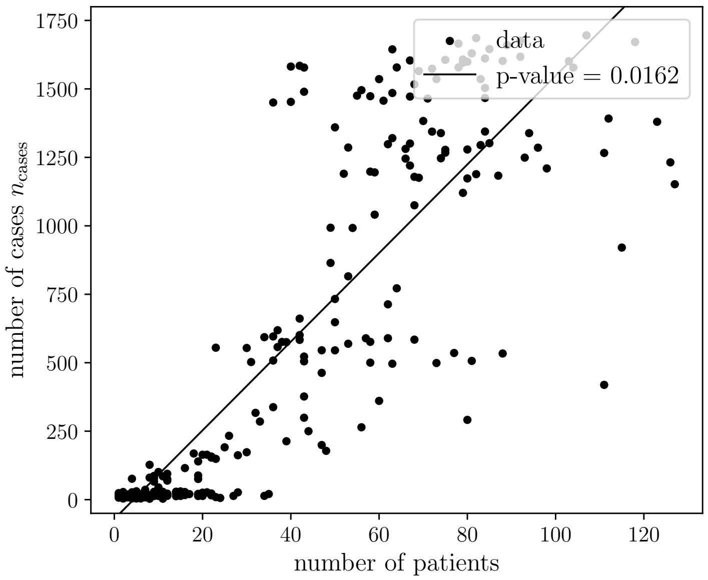

(b)

Figure S3. (A) Scatter plot of biweekly mean patient age and observed number of cases nationwide showing no significant relationship as given by p-value > 0.05 (B) Scatter plot of biweekly number of confirmed positive RHUH patients and observed number of cases nationwide showing a clear positive value that is significant as given by p-value < 0.05

S.2. Deep learning model architecture

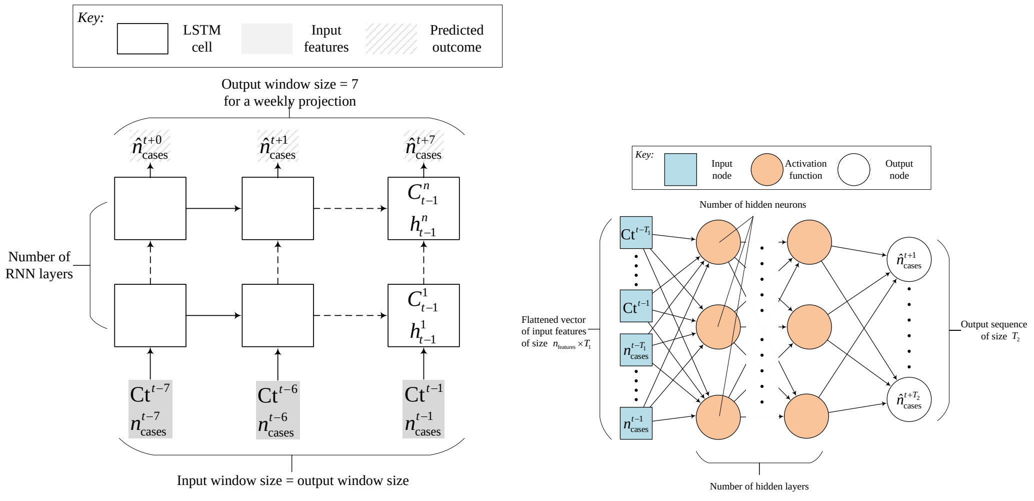

(a) **(b)**

Figure S4. Model architecture of the a) stacked LSTM (SEQ) and the (b) feedforward neural network (DNN) models.

S.3. Effect of additional training data on model performance

The model development methodology in Section 3.3 was applied to the entire patient cohort of RHUH (Figure 3A). Groups 1 and 2 were both used to train and cross-validate each model and its performance on Group 3 was tested using the mean squared error (MSE) criterion. The predictions of these models on the unseen data group (Group 3) are shown in Figure S5; the train and test errors are listed in Table S1, showing that the sequence-to-sequence (S2S) model outperforms all the other models as given by its low test error. This implies that deep learning model architecture can learn additional model representations as more training data becomes available. The distribution of the train and test errors is also shown by the box plots in Figure S7. The optimal hyperparameters of each model are listed in Table S2.

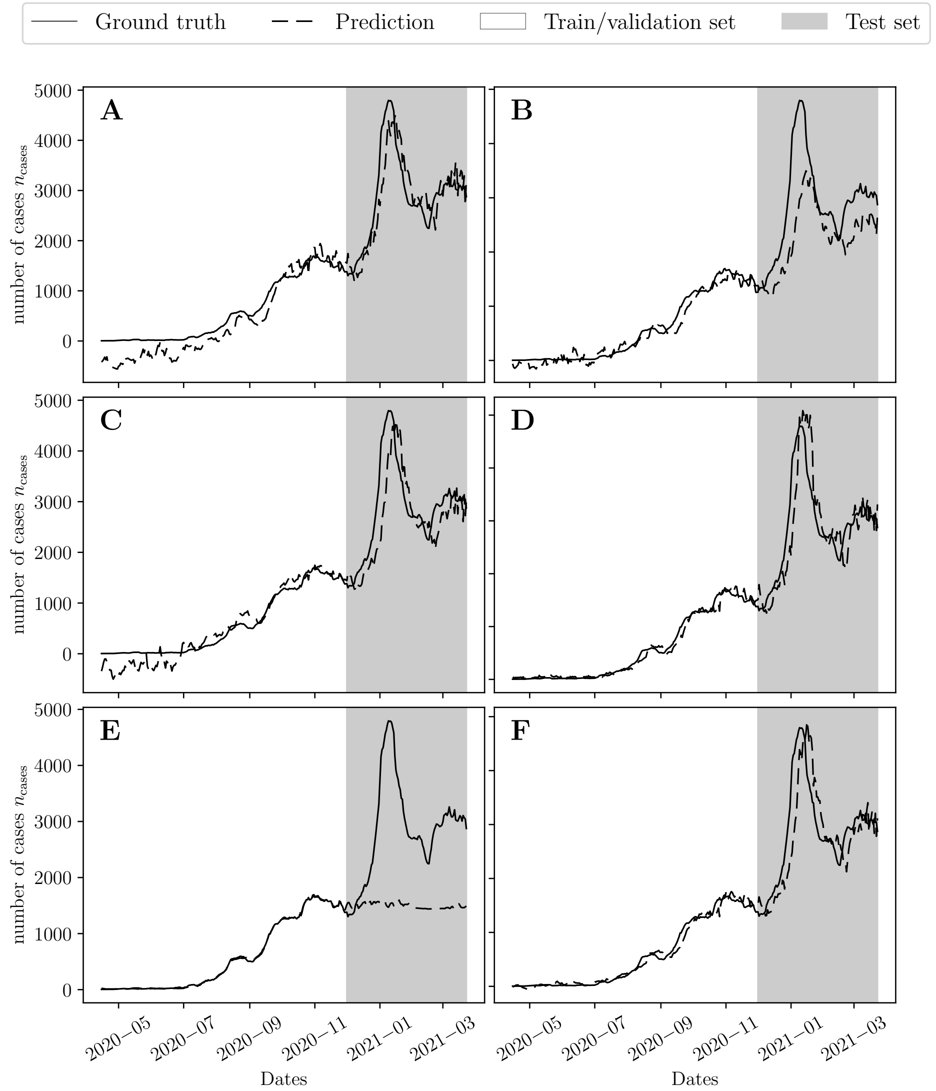

Figure S5. Predicted 7-day rolling average of daily number of cases on the unseen data group using (A) the sequence-to-sequence (S2S) model, (B) the stacked LSTM (SEQ), (C) The feedforward neural network (DNN), (D) The support vector machine regression (SVR) model, (E) The gradient boosting machine (GBM), and (F) the polynomial regression (OLS) model.
All models were tuned using the validation score of the combined discovery and test sets (Groups 1 and 2). The grey shaded region represents the unseen data group used to test the models' performance.

Table S1. Training and testing errors given by mean squared error (MSE) of different models constructed using the combined discovery and test sets (Groups 1 and 2).

**Model**	Train error	Test error
**Model**	Groups 1/2	Group 3
Sequence-to-sequence (S2S)	0.01460916	0.10580273
Stacked LSTM (SEQ)	0.00898817	0.34242207
Feedforward neural network (DNN)	0.01399668	0.20013593
Support vector machine regression (SVR)	0.00454633	0.13963704
Gradient boosting machine (GBM)	0.00035697	1.46476531
Polynomial regression (OLS)	0.00461187	0.16241863

The MSE in Equation (3) is computed using the standardized value of the predictions by normalizing them using the mean and standard deviation of all the daily number of cases (n_{cases}) given by 1221.7 and 1341.2, respectively.

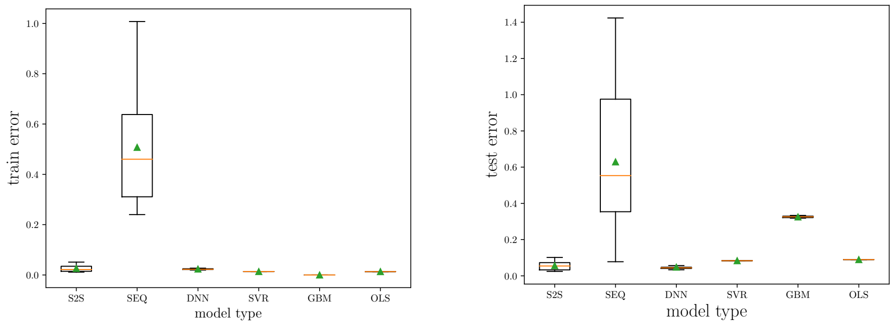

(a) **(b)**

Figure S6. Illustration of variance in a) training errors on the discovery group (Group 1) and b) test errors on the test group (Group 2) for different models. The errors were calculated using the MSE of the predicted and actual trajectories shown in Figure 5. The green triangles represent the mean error of 30 independent training runs for each model type. The orange lines represent the median error.

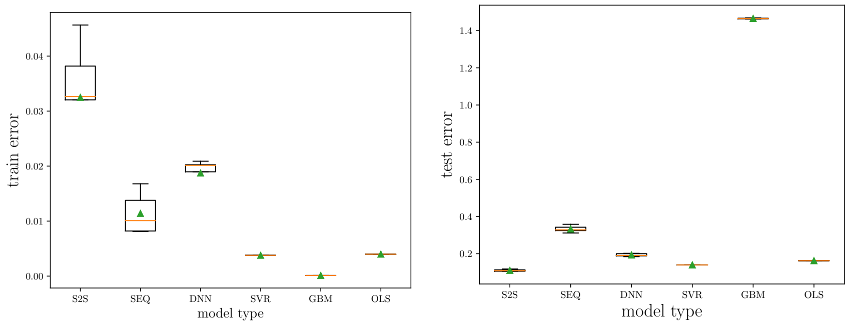

(a) **(b)**

Figure S7. Illustration of variance in a) training errors on combined discovery and test groups (Groups 1 and 2) and b) the test errors on the unseen group (Group 3) for different models. The errors were calculated using the MSE of the predicted and actual trajectories shown in Figure S5. The green triangles represent the mean error of 30 independent training runs for each model type. The orange lines represent the median error.

Table S2. Optimal hyperparameters of models developed using combined discovery and test groups (Groups 1 and 2).

Hyperparameter	Symbol	Value	Possible values
Sequence-to-sequence model (S2S)			
Sliding window size	T_1	6	1-40
Number of hidden neurons	n_{hidden}	1500	1-2500
Probability of dropout	P_{dropout}	0.0	0.0-0.9
Number of hidden layers	n_{hidden}	2	1-5
Teacher forcing probability	P_{teacher}	0.8	0.0-0.9
Learning rate	l_{rate}	1×10^{-4}	1×10^{-5}-1×10^{-2}
batch size	b_{size}	16	4-128
best epoch	$n_{\text{epochs}}^{\text{best}}$	16	1-n_{epochs}
Sequence completion model (SEQ)			
Number of hidden neurons	n_{hidden}	2500	1-2500
Probability of dropout	P_{dropout}	0.8	0.0-0.9
Number of hidden layers	n_{hidden}	2	1-5
Learning rate	l_{rate}	1×10^{-4}	1×10^{-5}-1×10^{-2}
batch size	b_{size}	32	4-128
best epoch	$n_{\text{epochs}}^{\text{best}}$	15	1-n_{epochs}
Deep neural network (DNN)			
Sliding window size	T_1	6	1-40
Number of hidden neurons	n_{hidden}	1500	1-2500
Probability of dropout	P_{dropout}	0.3	0.0-0.9
Number of hidden layers	n_{hidden}	1	1-5
Learning rate	l_{rate}	1×10^{-4}	1×10^{-5}-1×10^{-2}
batch size	b_{size}	4	4-128
best epoch	$n_{\text{epochs}}^{\text{best}}$	9	1-n_{epochs}
Support vector machine regression (SVR)			
Sliding window size	T_1	11	1-40
Ridge factor	λ	1×10^{-4}	1×10^{-3}-1.0
Margin of tolerance	ϵ	0.01	1×10^{-3}-1.0
Stopping criteria tolerance	ϵ_{tol}	0.1	1-5
Learning rate	l_{rate}	1×10^{-5}	1×10^{-5}-1×10^{-2}
Gradient boosting machine (GBM)			
Sliding window size	T_1	36	1-40
Subsample fraction	f_{sample}	0.9	0.1-1.0
Maximum portion of features	f_{features}	1.0	0.1-1.0
Decision tree maximum depth	D	2	1-5
Learning rate	l_{rate}	0.01	1×10^{-5}-1×10^{-2}
Maximum number of boosting stages	n_{stages}	3000	50-5000
Polynomial regression (OLS)			
Sliding window size	T_1	11	1-40
Ridge factor	λ	1×10^{-4}	1×10^{-3}-1.0
Degree	n_{degree}	1	1-5
Common fixed parameters			
Output window size (all models)	T_2	7	1-40
Maximum number of epochs (all models)	n_{epochs}	5000	
Kernel (SVR)		linear	
Early stopping patience (S2S,SEQ,DNN)	n_{patience}	200	
Optimizer (S2S,SEQ,DNN)		Adam	

The tuned hyperparameters of each model are reported underneath it. The fixed hyperparameters are reported at the bottom of the table.

S.4. Gaussian process model for RHUH patient cohort

We used the Gaussian process regression framework developed by [1] to reconstruct the pandemic trajectory in Lebanon. We used a grid search to tune the priors on all the viral kinetics model parameters and Gaussian process parameters ν and ρ, which control the bandwidth of the Gaussian kernel function. We attempted to minimize the MSE of the median predicted trajectory relative to the actual case counts and get a good estimate of the pandemic trajectory. We took the average of several runs to account for the randomness of Markov chain Monte Carlo (MCMC) sampling. Figure S8B shows the resulting predicted trajectory relative to the normalized case counts in Lebanon.

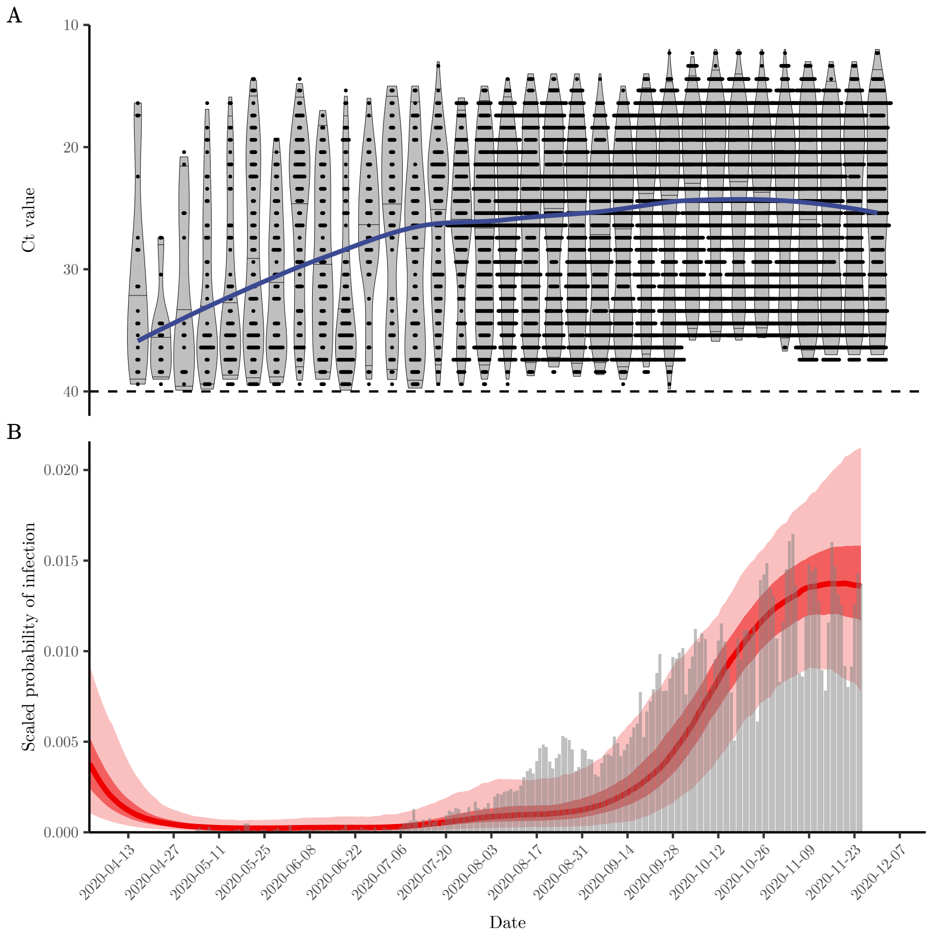

Figure S8. Incidence rate and pandemic trajectory predictions using the predictive framework developed by Hay et al. [1] (A) shows the cross-sectional Ct samples (violin plots) and smoothed average (solid blue line) obtained from RHUH throughout the pandemic in Lebanon. (B) Posterior distribution of relative probability of infection by date from a Gaussian process (GP) model fit to all observed Ct values (ribbons show 95% and 50% credible intervals, line shows posterior median). The y-axis shows relative rather than absolute probability of infection, as the underlying incidence curve must sum to one. The grey bars show the true case counts in Lebanon from the start of infection and have been normalized by the total number of cases observed in Lebanon throughout the observation time period shown (March 01, 2020 through November 30, 2020).

S.5. Inferring the pandemic trajectory in Massachusetts using Brigham and Women's Hospital cross-sectional Ct data

Data collected from BWH by [1] was used to test the performance of the models developed in this paper. Figure S10 shows the predicted incidence rates based on the Ct values observed at BWH (shown in Figure S9A). The deep learning models (Figures S10A, B, and C) had less accuracy than both SVR and OLS models (Figures S10D and F). Slightly biasing the Ct values resulted in better performance of all deep learning models (not shown in this paper), implying that they are very sensitive to fluctuations in Ct, which could be due to slightly different PCR machine calibration and specimen collection methods.

The SVR model shows very good performance on the BWH dataset but we advise caution when using such predictive models without prior cross-validation as is being done in this section.

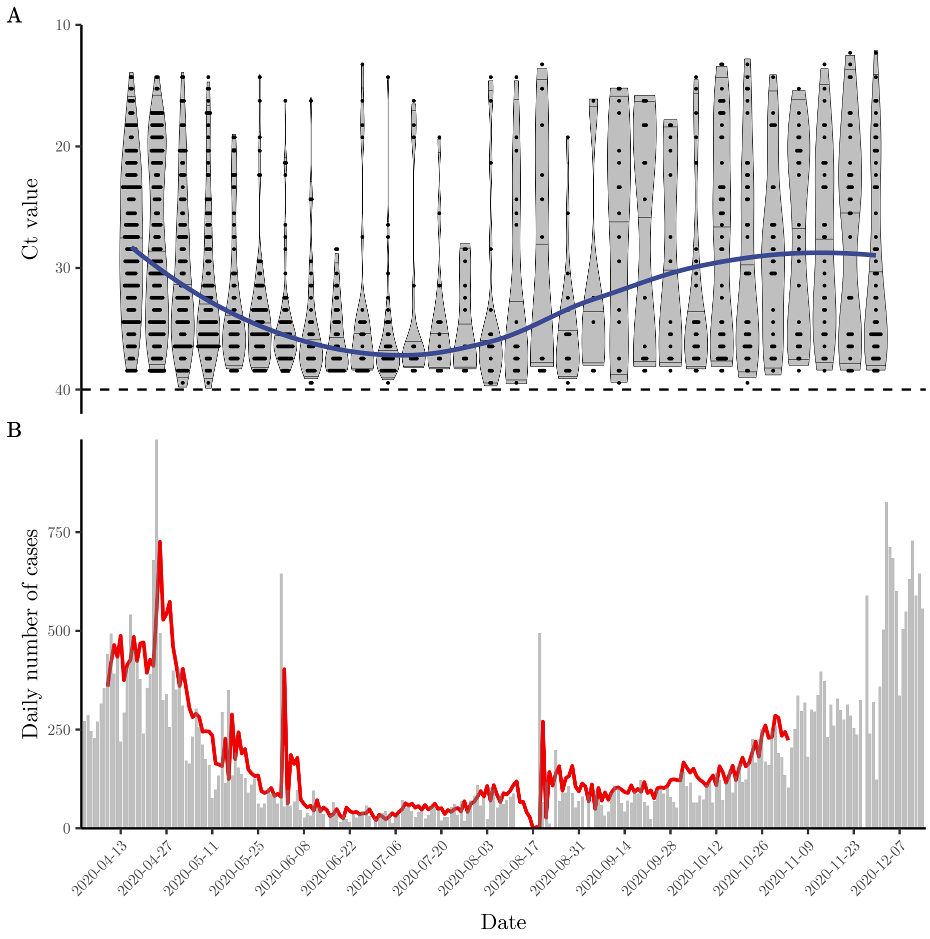

Figure S9. Incidence rate and pandemic trajectory predictions using the support vector machine regression (SVR) model (A) shows the cross-sectional Ct samples (violin plots) and smoothed average (solid blue line) obtained from Brigham and Women's Hospital (BWH) throughout the pandemic in Massachusetts. (B) Predicted pandemic trajectory of the SVR model fit to all observed Ct values. The grey bars show the true case counts in Massachusetts from the start of infection.

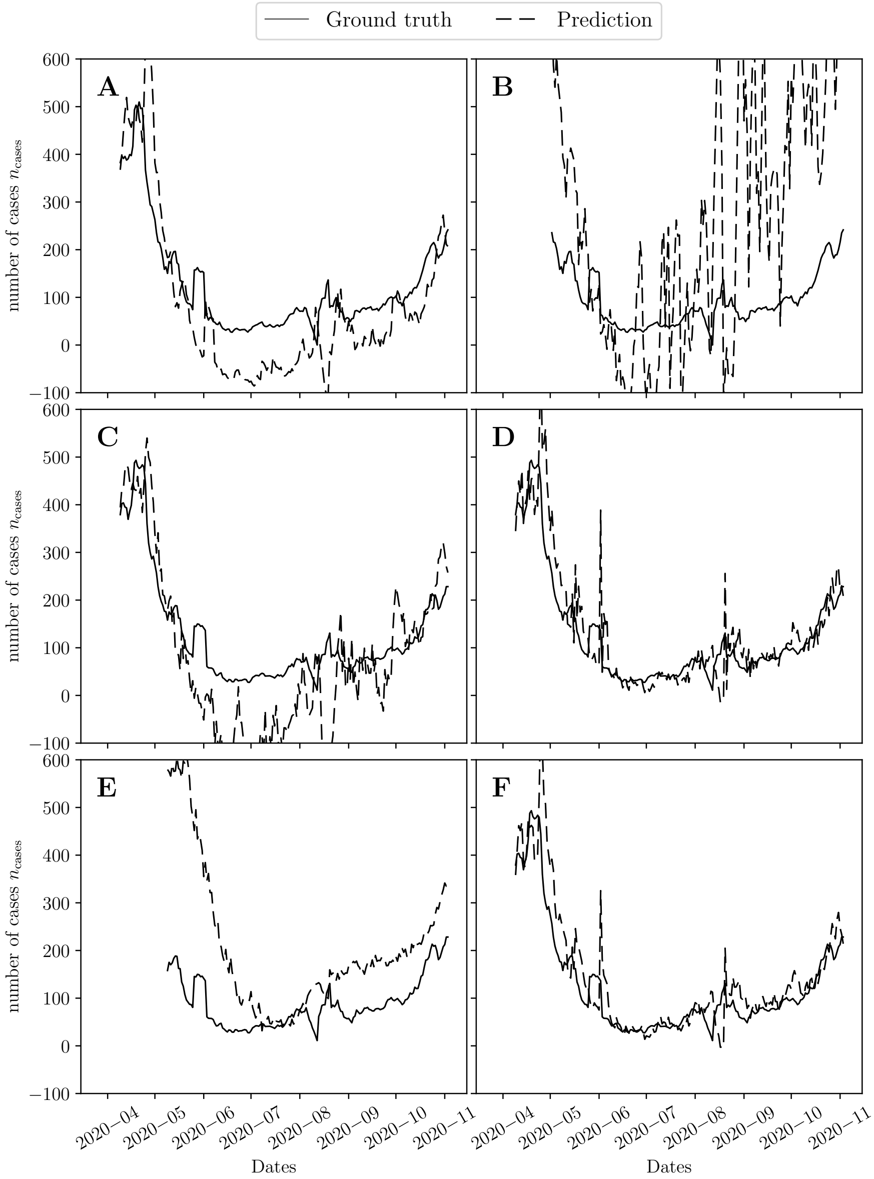

Figure S10. Predicted 7-day rolling average of the daily number of cases in Massachusetts predicted using (A) the sequence-to-sequence (S2S) model, (B) the stacked LSTM (SEQ), (C) The feedforward neural network (DNN), (D) The support vector machine regression (SVR) model, (E) The gradient boosting machine (GBM), and (F) the polynomial regression (OLS) model. The Ct values used in inference were obtained from Brigham and Women's Hospital (BWH) [1].

S.6. Deployment of the predictive model

We deployed the S2S model developed using the entire dataset (see supplementary material Section S.3) in a user-friendly interface and made it publicly available through https://covid-forecaster-lebanon.herokuapp.com [2]. The user interface allows the user to enter the number of cases and Ct values observed for a certain number of days backward (which represents the optimal sliding window obtained through hyperparameter tuning). The S2S model is used to infer the predicted total number of cases for the coming week (i.e., the average predicted case counts multiplied by 7). The data can be entered manually or copied from a spreadsheet. Continuous updates and patches will be applied to the dashboard to incorporate all the other models and provide additional visuals.

References

1. Hay, J.A.; Kennedy-Shaffer, L.; Kanjilal, S.; Lennon, H.J.; Gabriel, S.B.; Lipsitch, M.; Mina, M.J. Estimating epidemiologic dynamics from cross-sectional viral load distributions. *Science* **2021**, p. eabh0635. doi:10.1126/science.abh0635.
2. COVID-19 weekly forecaster. https://covid-forecaster-lebanon.herokuapp.com. [Online; accessed 31-March-2022]
